# Perception in context of Chinese and Japanese: the role of language proficiency

**DOI:** 10.3389/fpsyg.2025.1528955

**Published:** 2025-01-23

**Authors:** Sa Lu, Rongxia Ren, Ting Guo, Xiaoyu Tang

**Affiliations:** ^1^Research Center of Language and Cognition, School of Foreign Languages, Ningbo University of Technology, Ningbo, China; ^2^School of Education Science, Yan’an University, Yan’an, China; ^3^School of Psychology, Liaoning Collaborative Innovation Center of Children and Adolescents Healthy Personality Assessment and Cultivation, Liaoning Normal University, Dalian, China

**Keywords:** language context, language proficiency, phonetic perception, Chinese-Japanese bilinguals, vowel judgment

## Abstract

**Introduction:**

The effect of language context on bilinguals has been studied in phonetic production. However, it is still unclear how the language context affects phonetic perception as the level of second language (L2) proficiency increases.

**Methods:**

Chinese–Japanese auditory cognates were selected to avoid the interference of semantics and font or spelling processing. Low- to high-proficiency Chinese–Japanese bilinguals, as well as Chinese and Japanese monolinguals, were asked to judge whether the initial morpheme of the Chinese or Japanese words was pronounced with the vowels /a/ or /i/ in single- and mixed-language contexts.

**Results:**

The results found that low-proficiency bilinguals judged vowels faster in the single-language context than in the mixed-language context, whereas high-proficiency bilinguals showed no significant difference between the single- and mixed-language contexts.

**Discussion:**

These results indicate that as language proficiency increases, bilinguals appear to adaptively enhance phonetic perception when faced with different control demands in single-language and mixed-language contexts.

## 1 Introduction

The human brain has been equipped with a marked ability to acquire more than one language, as in bilingual individuals. Bilingual speakers are able to use and control each of their languages appropriately depending on the language context (Green and Abutalebi, 2013). Language context can be manipulated to explore the state of activation of the bilingual’s languages and language processing mechanisms at a given point in time and includes two typical language contexts, namely, a single language context and a mixed language context (Grosjean, 2001). Artificially creating a task-induced language context is a widely adopted method (Kałamała et al., 2022; Jiao et al., 2020a). For example, in the single-language context, words are presented in the same language, while words are presented interchangeably in two languages for a mixed-language context (Jiao et al., 2019). Adopting this method, many researchers have investigated the influence of language context on different levels of language processing, e.g., sentence, lexical, and phonetic processing (Fu et al., 2017; Jiao et al., 2020b; Rafeekh and Mishra, 2020; Timmer et al., 2019).

It has been found that phonetic processing is influenced by language context (Kuhl, 2004; Olson, 2013, 2015; Simonet, 2014). The acoustic parameters of native phonetic production are usually biased toward the second language (L2) in a mixed-language context than a single context as shown by acoustic analysis, such as the vowel height index (Simonet, 2014), voice onset time (Olson, 2013), pitch range and stressed vowel duration (Olson, 2015). In addition to phonetic production, phonetic processing also includes phonetic perception. However, it is still unclear how language contexts affect phonetic perception. Researchers propose that production and perception share representations and are thus strongly correlated (Best et al., 2001; Best and Tyler, 2007). There is some evidence for the involvement of partially overlapping frontal (i.e., Broca’s area) and posterior (i.e., Wernicke’s area) brain regions classically associated with production and perception, respectively, during perception and production (Agnew et al., 2013; Heim et al., 2003; Hickok and Poeppel, 2007; Price et al., 2011), lending further support to the idea of the interdependency of perception and production in the human brain. Hence, similar to phonetic production, we expect an effect of language context on phonetic perception.

Phonetic perception mainly focuses on whether individuals can successfully perceive differences in the pronunciation of sounds belonging to different language families (Isbell, 2016). The process of perception begins at the level of the sound signal and the process of audition. After processing the initial auditory signal, speech sounds are further processed to extract acoustic cues and phonetic information (Samuel, 2011). Experimental materials commonly used in phonetic research include word stimuli (e.g., /vanity/, Dong et al., 2005; Nakai et al., 2015), isolated syllabic stimuli (e.g., /ba/ /da/, Chen et al., 2008; Desmeules-Trudel and Joanisse, 2020), or isolated monophthong (e.g., /a/ /i/, Healy and Repp, 1982; Hewson-Stoate et al., 2006). In this research, we aim to investigate the influence of language context on phonetic perception. To create language context, we used word stimuli in the experiment. However, lexico-semantic activation could influence or bias speech perception, e.g., the well-noted Ganong effect. The “Ganong effect” is the tendency to perceive an ambiguous speech sound as a phoneme that would complete a real word rather than completing a nonsense/fake word (Ganong, 1980; Gianakas and Winn, 2016). For example, a sound that could be heard as either /g/ or /k/ is perceived as /g/ when followed by “ift” but perceived as /k/ when followed by “iss.” It is necessary to control the influence of semantic representation activated by word stimuli to investigate the effect of language context on phonetic perception.

To bypass this issue, the current study focuses on two logographic orthographies, namely, Chinese characters and Japanese Kanji. Indeed, Chinese and Japanese share many Chinese characters that have the same/similar orthography and meaning (i.e., Chinese-Japanese cognates), while their pronunciation is not always different (Nakayama, 2002). For example, the word “学校 (School)” is pronounced /Xue-Xiao/ in Chinese, but /Ga-Kko/ in Japanese; the word “优秀 (Excellent)” starts with the consonant /j/ in both Chinese “You-Xiu” and Japanese “Yu-Shu.” To investigate the influence of language context on phonetic perception while minimizing linguistic biases, we carefully selected auditory cognates with specific methodological considerations. We focused on vowels /a/ and /i/ due to their consistent pronunciation across Chinese and Japanese phonological systems. Both languages share these vowel sounds in their core vowel inventories: Japanese has five vowels (a, i, u, e, o), while Chinese has six vowels (a, o, e, i, u, ü). By selecting high-frequency, two-character words with identical or highly similar meanings, we aimed to minimize orthographic and semantic interference while maintaining linguistic authenticity. Notably, we deliberately excluded /u/−initial words due to their scarcity in both language corpora, which could introduce unintended variability in our stimuli set. This methodical approach to stimulus selection allows us to isolate the effects of language context on phonetic perception more precisely.

In addition to controlling the influence of semantic representation, the role of proficiency should also be considered. Some studies suggest that language proficiency may be an important key factor in shaping cross-language processing (Abutalebi et al., 2001; Abutalebi and Green, 2007). It was reported that proficient bilinguals show higher cognitive control than bilinguals with low language proficiency in a mixed-language context, but not in a single-language context (Hartanto et al., 2016; Ooi et al., 2018; Singh and Mishra, 2012; Ye et al., 2017). Thus, as L2 proficiency increases, how does bilinguals’ phonetic perception change in the single- and mixed-language contexts?

The goal of the present study was to examine the effect of language context on phonetic perception and how this effect was modulated by the Japanese proficiency of Chinese–Japanese bilinguals. The language context could be manipulated by changing the language families (i.e., Chinese and/or Japanese) in the oddball paradigm. The oddball paradigm was used to investigate the processing characteristics of auditory “odd” targets (Wottawa et al., 2022), which required standard (i.e., 80%), deviant (i.e., 10%), and target (i.e., 10%) categories of stimuli.

Participants in 4 groups of listeners (Chinese monolinguals, low-proficiency bilinguals, high-proficiency bilinguals, and Japanese monolinguals) performed a vowel judgment task in which they judged whether the initial morpheme of the Chinese or Japanese target word was pronounced with the vowel /a/ or /i/. Studies on vowel perception have found that vowels /a/ and /i/ in Chinese and Japanese are highly similar in perception (Chen et al., 2002; Wang and Deng, 2009). The task was performed in two contexts: (1) the single context, in which the standard and target stimuli were provided in the same language, either Chinese or Japanese, and (2) the mixed context, in which the standard stimuli were presented in Chinese (or Japanese) and the target stimuli in Japanese (or Chinese). We hypothesize that high-proficiency Chinese–Japanese bilinguals might judge vowels faster than low-proficiency bilinguals regardless of language context.

## 2 Methods

### 2.1 Materials and methods

#### 2.1.1 Participants

We conducted an *a priori* power analysis using G*Power 3.1 software to determine an appropriate sample size for our mixed-design experiment. Based on anticipated medium effect size (*f* = 0.25) for mixed ANOVA designs, with an alpha level of 0.05 and desired power of 0.80, the analysis suggested a total sample size of 64 participants would provide sufficient statistical power to detect significant interaction effects. Eighty undergraduates took part in the experiment. There were four groups, originally with 20 participants each. Four participants were excluded because their accuracy for targets were very low (lower than the threshold of −2.5 standard deviations (SDs) above the group mean). The final sample consisted of 76 participants (41 females; age range: 18–24 years; *M* = 20.80 years, SD = 1.49). The groups were as follows:

1. Chinese control group. This group consisted of 19 native speakers of Chinese (11 females; age range: 19–22 years; *M* = 19.79 years, SD = 0.92). They were recruited from Ningbo University of Technology with no Japanese background.
2. Low-proficiency Chinese–Japanese bilinguals. This group was made up of 19 first-year Japanese majors (13 females; age range: 18–21 years; *M* = 19.42 years, SD = 0.77) at Ningbo University of Technology. These participants were Chinese students who had no prior exposure to Japanese before entering the university and had completed only approximately 6 weeks of basic Japanese language courses. During this brief period, they had mastered only the fundamental pronunciation rules of Japanese kana, including the basic phonetic system and simple syllabic structures. Their limited language exposure and short-term learning experience distinguished them as low-proficiency bilinguals.
3. High-proficiency Chinese–Japanese bilinguals. The 19 participants (15 females; age range: 21–23 years; *M* = 22 years, SD = 0.58) in this group were Chinese and were fourth-year undergraduate students majoring in Japanese at Ningbo University of Technology. Before this experiment, their Japanese proficiency was rigorously tested and verified. Their proficiency was primarily indicated by the Test for Japanese Majors Band 4 (TJM4), a nationally recognized standardized test administered annually by the National Advisory Commission on Foreign Language Teaching in Higher Education in China. Additionally, all participants in this group had a minimum of three continuous years of intensive Japanese language study, extensive experience with Japanese language immersion, and demonstrated advanced comprehension and communication skills.
4. Japanese monolinguals. There were 17 males and 2 females, with ages ranging from 20 to 24 (*M* = 22 years; SD = 1.15). They were native speakers of Japanese and recruited from Okayama University with no background in Chinese at all.

In the present study, while Chinese monolinguals and Japanese monolinguals were unable to understand the meanings of the words in the other language, they were able to identify target vowels (either /a/ or /i/) based solely on auditory perception of phonemes. Both Chinese and Japanese languages have distinct vowel systems with overlapping features, which allowed participants to focus on the acoustic properties of the target vowels rather than relying on semantic or lexical knowledge.

The participants were free of head injury, and psychiatric disorders. None had any auditory or speech impairment, and all were right-handed, as handedness is known to correlate with the lateralization of phonological processing (Joanette et al., 1990). All gave voluntary consent for participation. The study was approved by the Ethics Committee of Ningbo University of Technology and Okayama University, and it was performed in accordance with the approved guidelines and the Declaration of Helsinki.

#### 2.1.2 Apparatus and stimuli

The stimulus materials consisted of two-character Chinese and Japanese auditory words that have the same font and meaning. The chosen two-character Japanese words need to be 2 kana and have dial, long, and promote tones. Dial tone is composed of consonant followed by a semivowel /y/ and a vowel, such as /Kyo-Ju/ (the meaning is “professor”). Long tone refers to lengthening the pronunciation of vowels by one beat, i.e., /toori/ (the meaning is “street”). Promote tone is a symbol used to express pause in Japanese, such as /Ke-Kka/ (the meaning is “result”).

To control for potential linguistic variations, we carefully balanced the target stimuli with initial vowels /a/ and /i/. These target words were selected from high-frequency corpora in both languages, ensuring comparable linguistic characteristics across experimental conditions. We employed a pseudo-randomized presentation order, strategically distributing target words to prevent clustering at the beginning or end of each experimental block.

The all the Chinese and Japanese auditory material was recorded by a female machine speaker in the Google vocabulary machine and was edited by Sound Engine software. The sampling rate was 44.1 kHz. The audios were used in the experiment .wav files. The average duration of the audio material was 700 ms (range: 550–850 ms). To control the influence of word frequency, high-frequency words were used in the experiment. According to the statistics of the National Institute of National Language Research in Japan,^1^ the top 5,000 words in terms of vocabulary use frequency are high-frequency words. In addition, words that appear 10 times per million are regarded as high-frequency words based on the statistics of the Institute of Language and Character Application of the Ministry of Education of China.^2^ Among the high-frequency words prescribed by the two languages, two-character words with the same meaning and font were selected as the auditory stimuli in this study. Eventually, 105 auditory words (two-character) were selected as the experimental stimuli. Twenty words were auditory target stimuli whose initials were pronounced with the vowels /a/ or /i/ in both Chinese and Japanese (e.g., 暗示, /An-Shi/ in Chinese, /An-Ji/ in Japanese). Another 85 words whose initials were pronounced with consonants (e.g., 决定, /Jue-Ding/ in Chinese, /Ke-Ttei/ in Japanese) were regarded as non-targets. For specific word materials, please refer to the Supplementary material.

#### 2.1.3 Procedure and design

The experiment was conducted in a dimly lit, sound-attenuated room. The experiment was presented with E-Prime software (1.1 version, Neurobehavioral Systems, Inc.), which controlled the presentation of the stimuli and the acquisition of data on a PC. Each trial began with the participant fixating on a white cross in the center of the screen for 800 ms. With the white cross turning gray, a 550–850 ms auditory stimulus was presented via headphones. Finally, the gray cross turned white for 2,000 ms to allow the participant to make responses. The participants were instructed to judge whether the initial morpheme of the Chinese or Japanese words was pronounced with the vowels /a/ or /i/. For example, when hearing Japanese words An-Zen (safety) or I-Ken (idea), click the left mouse button; when hearing Ai-Qing (love) or Yi-Yi (meaning), click the left mouse button; otherwise, do not click the mouse. As soon as the participant responded, the next trial began. However, if the participant did not respond within the given 2,000 ms, the experiment still continued with the next trial.

The experiment used a 4 (group: Chinese monolinguals, low-proficiency bilinguals, high-proficiency bilinguals, and Japanese monolinguals) × 2 (context: single- and mixed-language context) × 2 (target language: Japanese and Chinese) three-factor mixed design, with the group as the between-participant factor and the context and target language as the within-participants factors. There were four separate blocks in the present study (see Table 1). The trials of each block were presented in a pseudorandom order. The target items were not allowed to be repeated in consecutive trials. Each block contained 115 auditory stimuli presentations, including 30 target words and 85 nontarget words.

**Table 1:** Experimental conditions and examples of words used in the study.

Task	Context	Standard	Deviant	Target
Judging Chinese targets	Single	Chinese	None	Chinese
		/Ren-Sheng/ (Life)		/Ai-Qing/ (Love)
	Mixed	Japanese	Chinese	Chinese
		/Ho-Ho/ (Method)	/Lv-Xing/ (Travel)	/Yi-Si/ (Meaning)
Judging Japanese targets	Single	Japanese	None	Japanese
		/Ke-Kka/ (Reason)		/In-Yo/ (Quote)
	Mixed	Chinese	Japanese	Japanese
		/Ping-Jia/ (Assessment)	/Sa-Kka/ (Writer)	/Ai-Ko/ (Hobby)

The targets were the words for which the initial morpheme was /a/ or /i/. The standard and deviant stimuli were words whose initial morpheme begins with a consonant. / / represents the pronunciation of Chinese or Japanese words. () represents the semantics of Chinese or Japanese words.

Specifically, in single context blocks, both 85 standard and 30 target stimuli were Chinese words, i.e., single-CC; both 85 standard and 30 target stimuli were Japanese words, i.e., single-JJ. In mixed context blocks, deviant stimuli were added to prevent participants from employing processing methods for “oddball” stimuli. The 70 standard stimuli were Japanese words, and the 15 deviant and 30 target stimuli were Chinese words, namely, mixed-JC; the 70 standard stimuli were Chinese words, and the 15 deviant and 30 target stimuli were Japanese words, namely, mixed-CJ.

**Figure 1:**
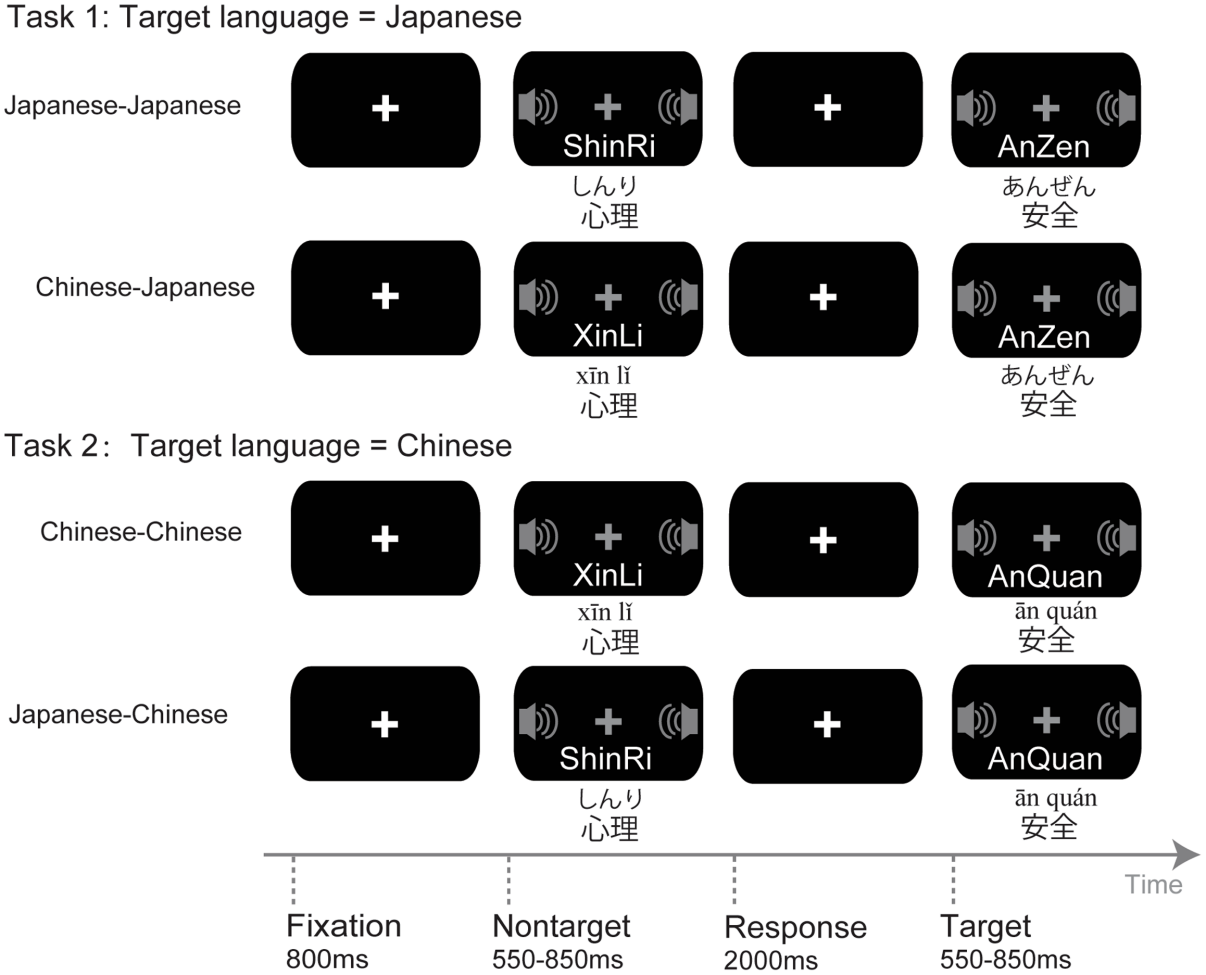
Experimental procedure. An illustration of conditions in the experiment.

Following one practice block, each participant completed 4 experimental blocks. The blocks were counterbalanced across Chinese and Japanese participants. Chinese participants (Chinese monolinguals, low-proficiency bilinguals, high-proficiency bilinguals) completed single-CC, mixed-JC, mixed-CJ, and single-JJ blocks in turn. The Japanese monolinguals successively completed single-JJ, mixed-CJ, mixed-JC, and single-CC blocks. Each block took approximately 6 min, with rest breaks given at the end of each block (Figure 1).

### 2.2 Data analysis

The analyzes for the two dependent variables, reaction times (RT), and accuracy of targets were conducted separately and implemented in Rstudio (RStudio Team, 2021) using the lme4 package (Bates et al., 2015) and bruceR package (Bao, 2022). Linear mixed effects models were used because they can account for variability in the results attributed to individual participants and items (Baayen, 2008).

The RT data were submitted to linear mixed-effects models, and the accuracy data were submitted to generalized mixed-effects models. The models included the fixed effects of group (Chinese monolinguals, low proficiency bilinguals, high proficiency bilinguals, and Japanese monolinguals), context (single and mixed language context), target language (Japanese and Chinese), and their interactions as fixed effects and the random intercepts capturing the differences across subjects and items. We assessed the contribution of each random slope to each model using likelihood-ratio tests and reported the best-fitting model justified by the data. The factors were sum coded as follows: context (single =0.5, mixed = −0.5) and target language (Chinese =0.5, Japanese = −0.5). The statistical significance of the main effects and interactions were judged based on *p* values (*p* < 0.05).

We calculated the mean response times (RTs) for target correct responses for each participant and condition. First, missed trials, representing 6.27% of the data, were removed from the analysis. Second, trials for which response times were above or below 2.5 SDs from the participants’ means, representing 2.43% of the data, were eliminated (Dijkstra et al., 2015; Van Ginkel and Dijkstra, 2020). A total of 8.70% of the trials were discarded from RT analysis. As Reaction times were not normally distributed, they were log-transformed. RTs were positively skewed (skewness = 0.71, D = 0.047, *p* < 0.001) and therefore were log-transformed for use as the dependent variable in a mixed-effects model.

## 3 Results

### 3.1 Reaction times

**Figure 2:**
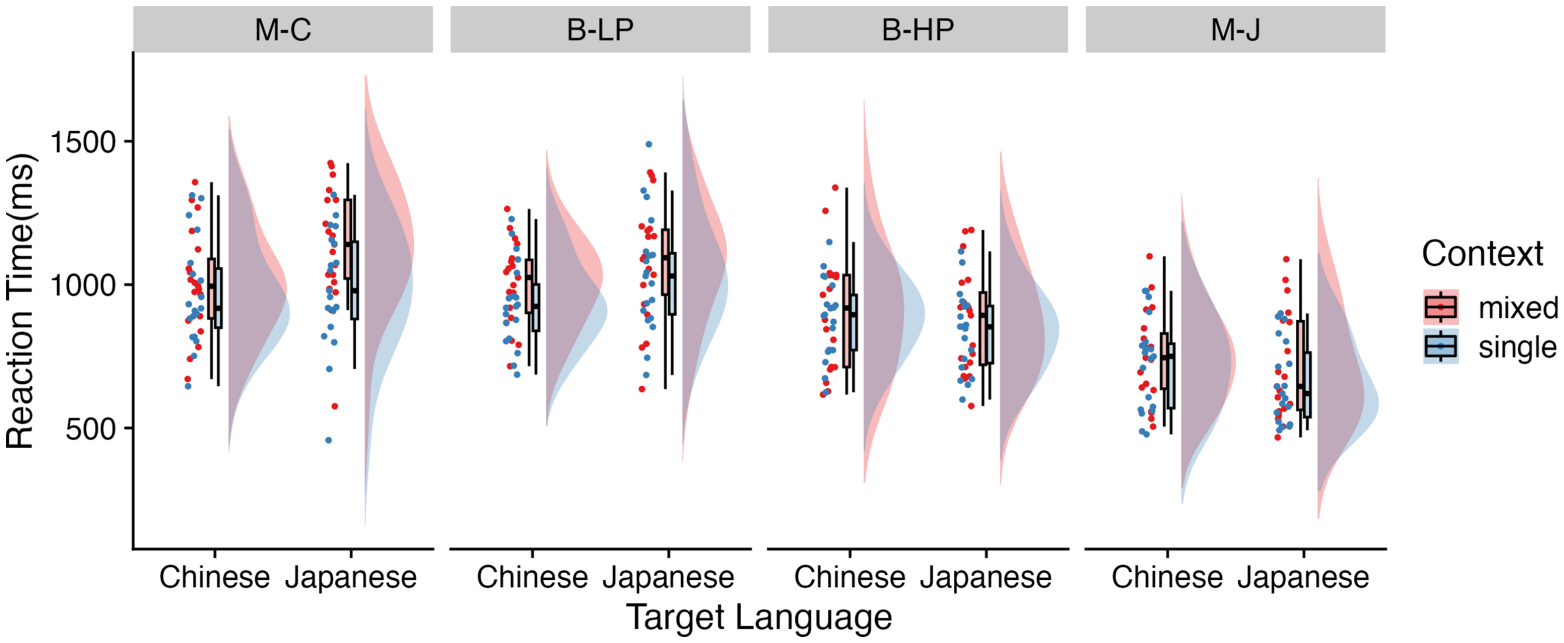
Raincloud plots showing the RTs in the vowel judgment task for each language context for each target language type (Chinese and Japanese trials) among four groups. M-C represents Chinese monolinguals, B-LP represents low proficiency bilinguals, B-HP represents high proficiency bilinguals, and M-J represents Japanese monolinguals.

Table 2 presents the average LogRT for correct responses per condition, Figure 2 presents the average RT for correct responses per condition, and the outcome of the linear mixed effects analysis on the LogRT data is provided in Table 3. The LogRT data were submitted to a liner mixed-effects model, with context, target language, group, and their interactions as fixed effects. Subjects and items were simultaneously included as crossed random effects, with the by-subject random slopes for context and target language, and by-item random slopes for context.

**Table 2:** Mean ± standard deviation (SD) of LogRT for all experimental conditions and statistical value of comparison between single and mixed language contexts.

Group	Target language	Context			
		Single	Mixed	*t*	*p*
M-C	Japanese	6.81 ± 0.33	6.96 ± 0.30	−6.47^***^	<0.001
	Chinese	6.82 ± 0.24	6.86 ± 0.26	−1.47	=0.145
B-LP	Japanese	6.85 ± 0.03	6.92 ± 0.30	−2.81^**^	=0.006
	Chinese	6.78 ± 0.25	6.86 ± 0.25	−3.14^**^	=0.002
B-HP	Japanese	6.66 ± 0.27	6.70 ± 0.28	−1.69	=0.094
	Chinese	6.72 ± 0.23	6.74 ± 0.28	−0.84	=0.404
M-J	Japanese	6.43 ± 0.29	6.50 ± 0.31	−3.18^**^	=0.002
	Chinese	6.50 ± 0.31	6.54 ± 0.32	−1.86	=0.066

M-C represents Chinese monolinguals, B-LP represents low proficiency bilinguals, B-HP represents high proficiency bilinguals, and M-J represents Japanese monolinguals. ^*^ represents *p* < 0.05; ^**^ represents *p* < 0.01; ^***^ represents *p* < 0.001.

**Table 3:** Estimates, standard errors, and *t* values for the fixed effects of the linear mixed effect model for LogRT.

	Estimate	SE	*t*	*p*
(Intercept)	6.87	0.04	164.16^***^	<0.001
Context (mixed vs. single)	0.09	0.02	4.56^***^	<0.001
Language (Chinese vs. Japanese)	0.05	0.03	1.76	=0.08
Group 2	−0.01	0.06	−0.23	=0.82
Group 3	−0.16	0.06	−2.80^*^	=0.01
Group 4	−0.37	0.06	−6.33^***^	<0.001
Context × Language	0.11	0.02	5.81^***^	<0.001
Context × Group 2	−0.02	0.03	−0.73	=0.47
Context × Group 3	−0.06	0.03	−2.26^*^	=0.03
Context × Group 4	−0.03	0.03	−1.23	=0.22
Language × Group 2	0.02	0.04	0.65	=0.52
Language × Group 3	−0.11	0.04	−3.19^***^	<0.001
Language × Group 4	−0.11	0.04	−3.08^***^	<0.001
Context × Language × Group 2	−0.13	0.03	−4.83^***^	<0.001
Context × Language × Group 3	−0.10	0.03	−3.72^***^	<0.001
Context × Language × Group 3	−0.08	0.03	−3.31^***^	<0.001

Group 2 represents low proficiency bilinguals, Group 3 represents high proficiency bilinguals, and Group 4 represents Japanese monolinguals. ^*^ represents *p* < 0.05; ^**^ represents *p* < 0.01; ^***^ represents *p* < 0.001.

As seen in Table 3, the main effects of context were significant, with slower responses for mixed context trials (*M* = 906 ms, SD = 225 ms) than single context trials (*M* = 846 ms, SD = 266, *t* = 4.56, *p* < 0.001, Cohen’s *d* = 0.21). Then, the effect of Group variable was significant, indicating that the RTs in the Japanese monolinguals (*M* = 694 ms, SD = 231) were shorter than the Chinese monolinguals (*M* = 994 ms, SD = 278, *t* = 6.17, *p* < 0.001, Cohen’s *d =* 0.87), the low-proficiency bilinguals (*M* = 959 ms, SD = 243, *t* = 5.94, *p* < 0.001, Cohen’s *d =* 0.71), and the high-proficiency bilinguals (*M* = 847 ms, SD = 235, *t* = 3.44, *p* = 0.006, Cohen’s *d =* 0.66). And, RTs in the high-proficiency bilinguals (*M* = 847 ms, SD = 235) were shorter than the Chinese monolinguals (*M* = 994 ms, SD = 278, *t* = 2.73, *p* = 0.047, Cohen’s *d =* 0.57). Statistically context × language × group, language × group, and context × language (β = 0.11, *SE* = 0.02, *t* = 5.81, *p* < 0.001) interactions were observed.

To disentangle the three-way interaction effect, we tested differences by using planned comparisons (see Figure 2). For Chinese monolinguals, the response was faster in the single language context (*M* = 954 ms, SD = 290) than in the mixed language context (*M* = 1,100 ms, SD = 304, *t* = −6.47, *p* < 0.001, Cohen’s *d = −*0.49) when judging Japanese (non-native) vowels. However, when judging Chinese (native) vowels, there was no significant difference to targets between the single language context (*M* = 942 ms, SD = 235) and mixed language context (*M* = 985 ms, SD = 255, *t* = −1.47, *p* = 0.14).

For Japanese monolinguals, the response was faster in the single language context (*M* = 649 ms, SD = 199) than in the mixed language context (*M* = 698 ms, SD = 235, *t* = −3.18, *p* = 0.002, Cohen’s *d = −*0.22) when judging Japanese vowels (native). However, when judging Chinese (non-native) vowels, there was no statistically significant difference between the single language context (*M* = 701 ms, SD = 236) and mixed language context (*M* = 729 ms, SD = 246, *t* = −1.86, *p* = 0.066).

For the low-proficiency bilinguals, participants responded significantly faster to the single language context than to the mixed language context regardless of whether they judged Japanese or Chinese vowels [Judging Japanese vowels: *M* (single context) = 992 ms, SD = 298, *M* (mixed context) = 1,060 ms, SD = 303, *t* = −2.82, *p* = 0.006, Cohen’s *d =* −0.23; Judging Chinese vowels: *M* (single context) = 908 ms, SD = 229, *M* (mixed context) = 978 ms, SD = 241, *t* = −3.37, *p* = 0.001, Cohen’s *d* = −0.3].

For the high-proficiency bilinguals, there was no significant difference between the single language context and the mixed language context regardless of judging Japanese or Chinese vowels [Judging Japanese vowels: *M* (single context) = 807 ms, SD = 221, *M* (mixed context) = 843 ms, SD = 245, *t* = −1.69, *p* = 0.094; Judging Chinese vowels: *M* (single context) = 854 ms, SD = 200, *M* (mixed context) = 884 ms, SD = 263, *t* = −0.84, *p* = 0.403].

### 3.2 Accuracy

Table 4 presents the accuracy of the targets based on the mean value for each condition across all participants. The accuracy data were submitted to a generalized mixed-effects model, with context, target language, group, and their interactions as fixed effects. Subjects and items were simultaneously included as crossed random effects, with the by-subject and by-item random slopes for context and target language. The fixed effects structure for the model of accuracy is summarized in Table 5.

**Table 4:** Mean ± standard deviation (SD) of accuracy for all experimental conditions and statistical value of comparison between single and mixed language contexts.

Group	Target language	Context			
		Single	Mixed	*z*	*p*
M-C	Japanese	0.88 ± 0.32	0.87 ± 0.34	0.20	=0.843
	Chinese	0.94 ± 0.24	0.97 ± 0.17	−1.69	=0.091
B-LP	Japanese	0.87 ± 0.33	0.91 ± 0.29	−1.71	=0.088
	Chinese	0.92 ± 0.27	0.91 ± 0.28	0.74	=0.458
B-HP	Japanese	0.98 ± 0.15	0.99 ± 0.08	−2.23^*^	=0.026
	Chinese	0.96 ± 0.19	0.97 ± 0.16	−0.25	=0.801
M-J	Japanese	0.98 ± 0.15	0.96 ± 0.20	1.05	=0.295
	Chinese	0.95 ± 0.22	0.93 ± 0.25	1.63	=0.104

M-C represents Chinese monolinguals, B-LP represents low proficiency bilinguals, B-HP represents high proficiency bilinguals, and M-J represents Japanese monolinguals. ^*^ represents *p* < 0.05; ^**^ represents *p* < 0.01; ^***^ represents *p* < 0.001.

**Table 5:** Estimates, standard errors, and *z* values for the fixed effects of the generalized linear mixed effect model for accuracy of targets.

	Estimate	SE	*z*	*p*
(Intercept)	3.00	0.23	12.91^***^	<0.001
Context (mixed vs. single)	0.28	0.22	1.25	=0.21
Language (Chinese vs. Japanese)	−1.31	0.31	−4.16^***^	<0.001
Group 2 (LP-B vs. C-M)	−0.33	0.29	−1.13	=0.26
Group 3 (HP-B vs. C-M)	1.47	0.33	4.43^***^	<0.001
Group 4 (J-M vs. C-M)	0.65	0.31	2.11^*^	=0.03
Context × Language	−0.66	0.45	−1.49	=0.14
Context × Group 2	−0.16	0.29	−0.54	=0.59
Context × Group 3	0.42	0.41	1.02	=0.31
Context × Group 4	−0.75	0.33	−2.27^*^	=0.02
Language × Group 2	1.02	0.34	2.97^***^	<0.001
Language × Group 3	2.13	0.45	4.74^***^	<0.001
Language × Group 4	2.04	0.38	5.34^***^	<0.001
Context × Language × Group 2	1.34	0.57	2.37^*^	=0.02
Context × Language × Group 3	1.85	0.81	2.30^*^	=0.02
Context × Language × Group 4	0.76	0.65	1.17	=0.24

Group 2 represents low proficiency bilinguals, Group 3 represents high proficiency bilinguals, and Group 4 represents Japanese monolinguals. ^*^ represents *p* < 0.05; ^**^ represents *p* < 0.01; ^***^ represents *p* < 0.001.

As shown in Table 5, the main effect of target language was significant, with higher accuracy for Chinese target trials (*M* = 0.94, SD = 0.23) than Japanese target trials (*M* = 0.93, SD = 0.26, *z* = −4.16, *p* < 0.001). Then, the effect of Group variable was significant, indicating that the accuracy in the low-proficiency bilinguals (*M* = 0.90, SD = 0.29) were lower than the high-proficiency bilinguals (*M* = 0.98, SD = 0.15, *z* = −5.94, *p* < 0.001), and the Japanese monolinguals (*M* = 0.95, SD = 0.21, *z* = −3.25, *p* = 0.007). And, accuracy in the high-proficiency bilinguals (*M* = 0.98, SD = 0.15) were higher than the Chinese monolinguals (*M* = 0.91, SD = 0.28, *z* = −4.43, *p* < 0.001). Statistically context × language × group, language × group, and context × group interactions were observed.

To disentangle the three-way interaction effect, we tested differences by using planned comparisons. For the high-proficiency bilinguals, there was a higher accuracy in mixed context (*M* = 0.99, SD = 0.08) than single context (*M* = 0.98, SD = 0.15, *z* = −2.23, *p* = 0.026) when judging Japanese vowels. However, there was no significant difference between single (*M* = 0.96, SD = 0.08) and mixed context (*M* = 0.97, SD = 0.15, *z* = −0.25, *p* = 0.801) when judging Chinese vowels. For Chinese monolinguals, low-proficiency bilinguals, and Japanese monolinguals, there was no significant difference between the single context and the mixed context regardless of judging Japanese or Chinese vowels (*ps* > 0.08; see Table 4 for detailed values).

## 4 Discussion

The present study investigated how language context affects phonetic perception performance in groups of Chinese monolinguals, Japanese monolinguals, and Chinese–Japanese bilinguals with different proficiency levels. Using Chinese–Japanese auditory cognates that shared the same orthography or meaning, we found that phonetic perception was affected by language context, which is consistent with phonetic production (Olson, 2013, 2015; Simonet, 2014). Furthermore, the effect of language context on phonetic perception was modulated by language proficiency. Low-proficiency bilinguals could judge Japanese and Chinese vowels faster in a single-language context than in a mixed-language context. However, there were no differences for high-proficiency bilinguals between single- and mixed-language contexts in judging Japanese and Chinese vowels.

### 4.1 Monolingual group: the effect of language contexts on phonetic perception

In the study, Japanese and Chinese monolinguals judged Japanese vowels faster in a single language context than in a mixed language context. Two possible explanations are given in terms of language family and language environment.

First, Chinese and Japanese belong to different phonological systems (Wei, 2007; Shi, 2012). The Chinese phonetic system includes 24 vowels (6 monophthongs; 18 compound vowels, i.e., /an/, /ai/) and 22 consonants. Additionally, vowels and consonants can be pronounced separately. However, in the Japanese phonetic system, there are only 5 monophthongs and no independent consonants. Consonants are always combined with vowels. Therefore, the Japanese need to take the time to distinguish between Japanese vowels and consonants in the current vowel judgment task.

Second, given ambient linguistic diversity, monolinguals living in linguistically diverse communities regularly overhear languages that they neither understand nor speak, but this process may still promote new language learning (Bice and Kroll, 2019). Tracing this back to the source, it is found that there are Chinese students in the laboratory where Japanese monolinguals study. This might cause Japanese monolinguals to have greater exposure to Chinese. Therefore, when Japanese monolinguals judge Japanese vowels, unlike in the single context, Chinese words (implicit Chinese knowledge) in the mixed context produce interference, which results in slower responses and switch costs. Future research could recruit Japanese–Chinese bilinguals with different proficiency levels of Chinese and explore the influence of language context on phonetic perception.

### 4.2 Bilingual group: the effect of language contexts on phonetic perception

To our knowledge, this is the first study to investigate phonetic perception with Chinese–Japanese auditory words with the same font and meaning, which control the influence of semantic representation and font. Moreover, with the improvement of Japanese proficiency, bilinguals become more sensitive to Chinese and Japanese vowels. We found that low-proficiency bilinguals judged Japanese and Chinese vowels faster in a single-language context than in a mixed-language context. However, there were no significant differences for high-proficiency bilinguals between single- and mixed-language contexts in judging Japanese and Chinese vowels (see Table 2).

Bilinguals triggered different language control patterns to control the activation levels of two languages in accordance with language contexts (Green and Abutalebi, 2013). Bilinguals are able to establish normative phonological systems for their two languages (MacLeod and Stoel-Gammon, 2010), which could be different from monolingual norms (Flege and Eefting, 1987). Phonetic information was processed to match up with the phonological system of the particular language (Flege, 1995). Therefore, bilinguals could control the phonological systems between different languages in accordance with language contexts.

Low-proficiency bilinguals without enough Japanese experience could judge Japanese and Chinese vowels faster in a single-language context than in a mixed-language context. Considering the incomplete Japanese phonological system for low-proficiency bilinguals, the native phonetic processing strategy would be automatically activated (Thierry and Wu, 2004). Phonetic-processing strategies from their native language inevitably migrate to the processing of other languages (Sebastián-Gallés et al., 2005), which interferes with the two language systems. In a mixed-language context, low-proficiency bilinguals have to monitor the phonetic information of each auditory stimulus and then access the target language phonological system by inhibiting the activation of the native phonological system when judging Japanese vowels. In turn, low-proficiency bilinguals do not have any language switching demands in a single-language context, leading to more cognitive resources for vowel judgment. In short, low-proficiency bilinguals could not handle the extraction and conversion between the two phonological systems well in accordance with the current mixed-language context. Therefore, we observed that low-proficiency bilinguals were slower to judge vowels in a mixed-language context than in a single-language context.

For high-proficiency bilinguals, there were no differences between single- and mixed-language contexts in judging Japanese and Chinese vowels. Compared with low-proficiency bilinguals, high-proficiency bilinguals have a relatively complete Japanese phonological system and have advantages in interference suppression and cognitive flexibility due to the long-term experience with bilingual language (Costa and Santesteban, 2004; Price, 1999; Singh and Mishra, 2012, 2015). Whether in single- or mixed-language contexts, high-proficiency bilinguals could freely extract appropriate phonological systems for vowel discrimination. Thus—crucially—proficiency bilinguals could extract specific phonological systems to process phonetic information according to the language contexts.

In short, the present study shows the dynamic changes of the language control system with the different Japanese levels of Chinese–Japanese bilinguals. As language proficiency increases, bilinguals can be more flexible in adjusting the language control system according to the language context.

## 5 Limitations and future research

Several limitations should be noted. First, our study could not pinpoint whether English phonological awareness affects the current results of Chinese monolingual and Japanese monolingual participants. In China and Japan, all the students had 6 years of prior English instruction at the junior and senior high school levels. Considering the differences in English teaching between China and Japan, there may be differences in English phonological awareness between Chinese monolinguals and Japanese monolinguals.

Second, it is still unclear whether the phonological information provided by auditory stimuli would activate the semantic information of words in Chinese character and Kanji recognition. In the current research, we used Chinese-Japanese cognates to ensure the equivalence of semantics between the two languages. However, for bilinguals, the cognate words in this study may also have different strengths of lexical representation between Chinese and Japanese. Thus, whether asymmetrical semantic representation using cognate words affects Chinese and Japanese vowel perception needs further research.

Third, the logographic nature of Chinese and Japanese writing systems introduces unique challenges in cross-linguistic phonological processing. Unlike alphabetic languages that represent sounds through phonemic symbols, these languages employ characters that are inherently ideographic, representing semantic units with complex visual and linguistic properties. This fundamental difference in writing systems suggests that our findings may have limited direct transferability to bilingual populations using alphabetic writing systems. Future research should systematically explore how such writing system characteristics modulate phonological perception across different language families.

## 6 Summary

To conclude, our study sheds light on the flexibility of phonetic perception during different language contexts using the “oddball” paradigm, which is modulated by proficiency. Compared with the mixed-language contexts, low-proficiency Chinese–Japanese bilinguals respond faster in judging vowels at the phonetic perception level in a single-language context. However, for high-proficiency Chinese–Japanese bilinguals, there was no difference in judging vowels between the single- and mixed-language contexts. These results extend the literature on the role of language context in bilinguals’ phonetic perception. When faced with different control demands during single-language and mixed-language contexts, proficient bilinguals enhance phonetic perception by flexibly adjusting the language control system.

## Data Availability

The datasets presented in this study can be found in online repositories. The names of the repository/repositories and accession number(s) can be found here: https://osf.io/w76r5/.

## Footnotes

^1^ http://www2.kokken.go.jp/goityosa/index.html

^2^ http://corpus.china-language.edu.cn/
