# Supplementary material for: Perception in context of Chinese and Japanese: the role of language proficiency
Source: Front Psychol. 2025 Jan 23;16:1528955. doi: 10.3389/fpsyg.2025.1528955 (PMC11801191; doi:10.3389/fpsyg.2025.1528955)
Supplement: Supplementary file 1 [file Supplementary_file_1.docx]

**Supplementary Material**

| Nontarget | Chinese | Japanese | Nontarget | Chinese | Japanese | Nontarget | Chinese | Japanese |
| --- | --- | --- | --- | --- | --- | --- | --- | --- |
| 成功 | Cheng-Gong | Sei-Ko | 女性 | Nv-Xing | Jo-Sei | 加入 | Jia-Ru | Ka-Nyu |
| 宣传 | Xuan-Chuan | Sen-Den | 发行 | Fa-Xing | Ha-Kko | 企业 | Qi-Ye | Ki-Gyo |
| 倾向 | Qing-Xaing | Kei-Ko | 供给 | Gong-Ji | Kyo-Kyu | 学校 | Xue-Xiao | Ga-Kko |
| 强调 | Qiang-Diao | Kyo-Cho | 经验 | Jing-Yan | Kei-Ken | 患者 | Huan-Zhe | Kan-Sha |
| 形状 | Xing-Zhuang | Kei-Jo | 结婚 | Jie-Hun | Ke-Kkon | 决定 | Jue-Ding | Ke-Ttei |
| 通常 | Tong-Chang | Tsu-Jo | 效果 | Xiao-Guo | Ko-Ka | 工业 | Gong-Ye | Ko-Gyo |
| 勉强 | Mian-Qiang | Ben-Kyo | 作业 | Zuo-Ye | Sa-Gyo | 作家 | Zuo-Jia | Sa-Kka |
| 青春 | Qing-Chun | Sei-Shun | 事业 | Shi-Ye | Ji-Gyo | 资源 | Zi-Yuan | Shi-Gen |
| 实行 | Shi-Xing | Ji-Kko | 自然 | Zi-Ran | Shi-Zen | 实验 | Shi-Yan | Ji-Kken |
| 主张 | Zhu-Zhang | Shu-Cho | 脂肪 | Zhi-Fang | Shi-Bo | 社员 | She-Yuan | Sha-In |
| 人员 | Ren-Yuan | Jin-In | 周围 | Zhou-Wei | Shu-I | 修理 | Xiu-Li | Shu-Ri |
| 成果 | Cheng-Guo | Sei-Ka | 希望 | Xi-Wang | Ki-Bo | 条件 | Tiao-Jian | Jo-Ken |
| 生产 | Sheng-Chan | Sei-San | 事情 | Shi-Qing | Ji-Jo | 心理 | Xin-Li | Shin-Ri |
| 方向 | Fang-Xiang | Ho-Ko | 呼吸 | Hu-Xi | Ko-Kyu | 自由 | Zi-You | Ji-Yu |
| 上下 | Shang-Xia | Jo-Ge | 旅行 | Lv-Xing | Ryo-Ko | 精神 | Jing-Shen | Sei-Shin |
| 人生 | Ren-Sheng | Jin-Sei | 前后 | Qian-Hou | Zen-Go | 指挥 | Zhi-Hui | Shi-Ki |
| 当然 | Dang-Ran | To-Zen | 评价 | Ping-Jia | Hyo-Ka | 当初 | Dang-Chu | To-Sho |
| 平行 | Ping-Xing | Hei-Ko | 发展 | Fa-Zhan | Ha-Tten | 非常 | Fei-Chang | Hi-Jo |
| 防止 | Fang-Zhi | Bo-Shi | 故乡 | Gu-Xiang | Ko-Kyo | 平均 | Ping-Jun | Hei-Kin |
| 保证 | Bao-Zheng | Ho-Sho | 完全 | Wan-Quan | Kan-Zen | 个性 | Ge-Xing | Ko-Sei |
| 环境 | Huan-Jing | Kan-Kyo | 方针 | Fang-Zhen | Ho-Shin | 方法 | Fang-Fa | Ho-Ho |
| 政权 | Zheng-Quan | Sei-Ken | 保存 | Bao-Cun | Ho-Zon | 输入 | Shu-Ru | Yu-Nyu |
| 强化 | Qiang-Hua | Kyo-Ka | 预防 | Yu-Fang | Yo-Bo | 理由 | Li-You | Ri-Yu |
| 瞬间 | Shun-Jian | Shun-Kan | 选举 | Xuan-Ju | Sen-Kyo | 基本 | Ji-Ben | Ki-Hon |
| 成长 | Cheng-Zhang | Sei-Cho | 男女 | Nan-Nv | Dan-Jo | 结果 | Jie-Guo | Ke-Kka |
| 欢迎 | Huan-Ying | Kan-Gei | 今后 | Jin-Hou | Kon-Go | 将军 | Jiang-Jun | Sho-Gun |
| 形成 | Xing-Cheng | Kei-Sei | 教授 | Jiao-Shou | Kyo-Ju | 产业 | Chan-Ye | San-Gyo |
| 活性 | Huo-Xing | Ka-Ssei | 访问 | Fang-Wen | Ho-Mon | 优秀 | You-Xiu | Yu-Shu |
| 转换 | Zhuan-Huan | Ten-Kan |  |  |  |  |  |  |

**Table S1. List of nontarget auditory words**

**Table S2. List of target auditory words**

| Target | Chinese | Japanese | Target | Chinese | Japanese | Target | Chinese | Japanese |
| --- | --- | --- | --- | --- | --- | --- | --- | --- |
| 引用 | Yin-Yong | In-Yo | 以前 | Yi-Qian | I-Zen | 爱好 | Ai-Hao | Ai-Ko |
| 以下 | Yi-Xia | I-Ka | 以内 | Yi-Nei | I-Nai | 爱情 | Ai-Qing | Ai-Jo |
| 以后 | Yi-Hou | I-Go | 意见 | Yi-Jian | I-Ken | 安心 | An-Xin | An-Shin |
| 意义 | Yi-Yi | I-Gi | 遗传 | Yi-Chan | I-Den | 安全 | An-Quan | An-Zen |
| 意思 | Yi-Si | I-Shi | 医疗 | Yi-Liao | I-Ryo | 安定 | An-Ding | An-Tei |
| 意图 | Yi-Tu | I-To | 一般 | Yi-Ban | I-Ppan | 暗示 | An-Shi | An-Ji |
| 以上 | Yi-Shang | I-Jo | 印象 | Yin-Xiang | In-Sho |  |  |  |
